# Extended Subaperture Imaging Method for Airborne Low Frequency Ultrawideband SAR Data

**DOI:** 10.3390/s19204516

**Published:** 2019-10-17

**Authors:** Daoxiang An, Wu Wang, Leping Chen

**Affiliations:** College of Electronic Science and Technology, National University of Defense Technology, Changsha 410073, China; daoxiangan@nudt.edu.cn (D.A.); gfkdclp@126.com (L.C.)

**Keywords:** low frequency, UWB SAR, subaperture imaging, azimuth ambiguities

## Abstract

The subaperture processing is one of the essential strategies for low frequency ultrawideband synthetic aperture radar (LF UWB SAR) imaging, especially for the real-time LF UWB SAR imaging because it can improve the parallelization of the imaging algorithm. However, due to the longer synthetic aperture of LF UWB SAR, the traditional subaperture imaging encounters an azimuth ambiguities problem, which severely degrades the focused quality of the imaging results. In this paper, the reason for the presence of azimuth ambiguities in the LF UWB SAR subaperture imaging and its influence on image quality is first analyzed in theory. Then, an extended subaperture imaging method based on the extension of subaperture length before Range Cell Migration Correction (RCMC) was proposed. By lengthening the subaperture length, the azimuth ambiguities are effectively eliminated. Finally, the extended part of subaperture is wiped off before the azimuth compression (AC), and the LF UWB SAR image of high focused quality is obtained. The correctness of the theory analysis and the effectiveness of the proposed method have been validated through simulated and real LF UWB SAR data.

## 1. Introduction

The low frequency ultra wideband synthetic aperture radar (LF UWB SAR) has excellent foliage- or ground-penetrating capability to detect concealed targets [1,2,3,4]. However, the UWB signal and large integration angle used in low frequency (<1 GHz) UWB SAR bring new complexities and challenges to the traditional SAR image formation processing. In the low frequency ultrawideband synthetic aperture radar (LF UWB SAR) real-time imaging, taking into account the computational load, storage space, and real-time requirements, it is usually to adopt the subaperture imaging strategy [5,6,7,8,9,10,11,12], which could greatly improve the real-time imaging performance. For example, in [5], Moreira proposed the subaperture approach for real-time, which does not make use of the FFT and requires a reduced number of subapertures to achieve the desired geometric resolution. The method is performed in a time-domain, and it is not easy to be integrated with the motion compensation (MOCO) method, especially the kinds of autofocus methods based on raw data. Thus, it cannot be directly applied to the real-time imaging of airborne SAR. Similarly, Sun proposed a real-time imaging algorithm based on subaperture chirp scaling dechirp in [9]. In [10], a modified subaperture imaging algorithm is proposed for high-squint-mode synthetic aperture radar (SAR) mounted on maneuvering platforms for range walk correction.

However, the traditional subaperture imaging has severe influences on the obtained imaging results for the appearance of the azimuth ambiguity. Aiming at this problem, this paper conducts an in-depth analysis on the problem of the azimuth ambiguities induced by the azimuth subaperture imaging processing for LF UWB SAR data, and an extended azimuth subaperture imaging approach without the azimuth ambiguities problem was proposed.

In some special SAR systems, such as the spaceborne spotlight SAR, the Doppler bandwidth is usually higher than the pulse repeat frequency (PRF) to achieve the large swath [13,14]. However, this under-sampling makes the signals aliased along the azimuth direction, and the ambiguities will be displaced in azimuth [13,14,15]. Up to now, several methods have already been proposed for reducing the azimuth ambiguities. In [13], an ideal filter concept for removal of amplitude and phase errors of impulse response function (IRF) is adapted to suppress azimuth ambiguities. In [14], a preprocessing operation was firstly performed to eliminate the azimuth ambiguities phenomenon before applying the following imaging processing.

Different from the azimuth ambiguity mentioned above caused by the under-sampling in the slow-time domain, the azimuth ambiguity problem discussed in this paper is not caused by the system under-sampling. In our discussion, the system PRF is higher than the Doppler bandwidth, and the azimuth ambiguities are caused by the under-sampling in the azimuth Doppler domain. This type of azimuth ambiguity exists in both full aperture imaging processing and subaperture imaging processing, and it is more severe in the subaperture imaging processing. The azimuth ambiguities discussed in this paper have rarely been studied in previous SAR literature. In [16], the study of azimuth ambiguities in subaperture Nonlinear Chirp Scaling (NCS) algorithm is presented, which points out that the azimuth ambiguities exist in the subaperture imaging for LF UWB SAR, but it did not give an effective method to resolve the azimuth ambiguities problem.

In the Fourier transform based imaging algorithms, the azimuth extension of imaging result is determined by the sampling interval of Doppler frequency and is equal to the length of aperture. However, in practice, the azimuth extension of scene illuminated by the radar beam is larger than the length of aperture, which will induce the azimuth ambiguities in imaging result. In the subaperture imaging, every subaperture image is contaminated by this type of azimuth ambiguity. After combining them into the full aperture image, the quality of imaging result is severely degraded.

Based on the above analysis, to suppress the azimuth ambiguities, the azimuth extension of imaging result should be enlarged, which can be done by reducing the sampling interval of Doppler frequency. One solution to achieve this purpose is increasing the length of subaperture by zero-padding, namely, the extension of subaperture length.

Based on the previous work, this paper carries out an in-depth theory analysis on this type of azimuth ambiguities, and a novel method was proposed based on the extension of subaperture length before Range Cell Migration Correction (RCMC) processing to suppress the azimuth ambiguities. Theory analysis and experiment results show that, when the subaperture is extended twice in length, the influence of azimuth ambiguities will be effectively suppressed. This paper is organized as follows. Section 2 presents the traditional subaperture imaging processing for LF UWB SAR. Section 3 introduces the principle of azimuth ambiguities. In addition, the analysis begins with discrete format of the imaging procedure; the azimuth ambiguities in the full aperture imaging and the subaperture imaging are also addressed in this section. The proposed extended subaperture method for the azimuth ambiguity suppression is presented in Section 4. Then, the simulated and real data experimental results are shown in Section 5. Finally, conclusions are given in Section 6.

## 2. Description of the Subaperture Imaging Processing

In the real data processing of LF UWB SAR with the long synthetic aperture, the subaperture method is always involved in imaging procedures to reduce the data volume and fulfill the memory requirements. In addition, the subaperture method can also improve the parallelism of the algorithm to meet the requirements of the real-time imaging processing.

**Figure 1:**
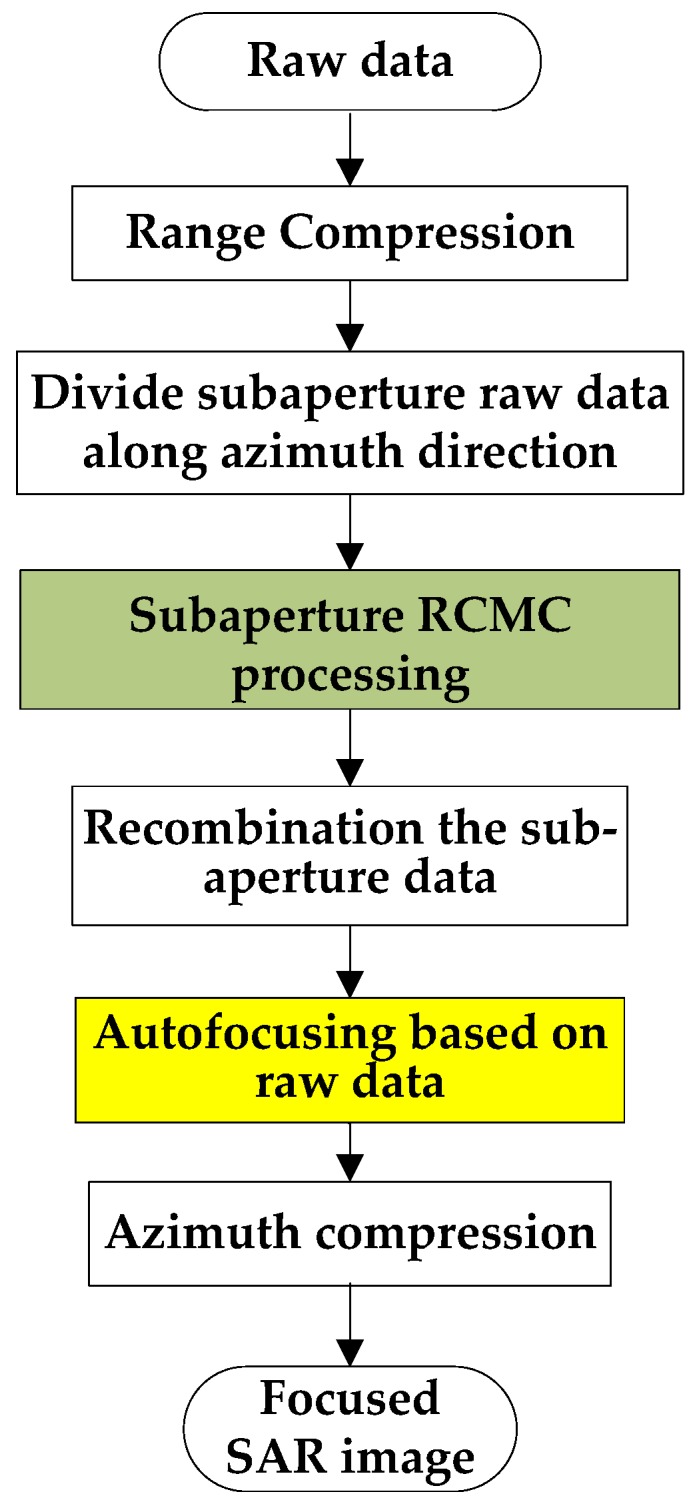
The flow diagram of traditional subaperture imaging processing for low frequency ultrawideband synthetic aperture radar (LF UWB SAR).

In the traditional subaperture imaging processing, the echo data after range compression (RC) is divided into subapertures along the azimuth direction. After that, the data of each block can be allocated to a single processor to perform the subaperture RCMC operation. Then, the subaperture echo data are re-combined to the full aperture data. Finally, the azimuth processing is performed, including the autofocusing and azimuth compression. Through the division of echo data, the entire data can be processed with the multicore processors, thereby enhancing the parallel of the algorithm. The subaperture RCMC processing can be performed by the frequency-domain algorithms, such as the Range Doppler (RD) algorithm [17], the Nonlinear Chirp Scaling (NCS) algorithm [4,16,18,19], the Extended Omega-K (EOK) algorithm [4], and so on. Figure 1 shows the flow diagram of the subaperture imaging processing for LF UWB SAR raw data. It should be noted that the operation of “autofocusing based on raw data” is an optional step, which is used for processing the real data of airborne SAR equipped with the low accuracy global navigation satellite system (GPSS) or inertial measurement unit (INS) data. To reduce the required memory and the computation load, the step of “Divide subaperture raw data along azimuth direction” was always performed after the range compression, which also could be performed before RC operation.

Neglecting the impact of envelope, the echo of a point target after demodulation can be expressed as

(1) $$ss\left(\tau ,\eta ;R_{T}\right)=\exp\left\{j\pi \gamma {\left[\tau -\frac{2R(\eta ;R_{T})}{c}\right]}^{2}\right\}\exp\left[-j\frac{4\pi f_{c}}{c}R\left(\eta ;R_{T}\right)\right],$$

where τ is the fast time, η is the slow time, $R_{T}$ is the closest range from the radar to the target, γ is the FM rate, $f_{c}$ is the center frequency, *c* is the speed of light, and $R(\eta ;R_{T})$ is the instantaneous slant range from a radar antenna phase center (APC) to the point target.

By applying the principle of stationary phase (POSP), the two-dimensional spectrum of Equation (1) is as follows:

(2) $$SS\left(f_{\tau },f_{\eta };R_{T}\right)=\exp\left(-j\pi \frac{f_{\tau }^{2}}{\gamma }\right)\exp\left(-j2\pi f_{\eta }\frac{X_{T}}{v}\right)\exp\left[-j\frac{4\pi R_{T}}{c}\sqrt{{\left(f_{c}+f_{\tau }\right)}^{2}-{\left(\frac{cf_{\eta }}{2v}\right)}^{2}}\right],$$

where $f_{\tau }$ is the range frequency. $f_{\eta }$ is the Doppler frequency, *v* is the velocity of platform, and $X_{T}$ is the azimuth location of the target.

Multiplying Equation (2) with function $H_{RC}\left(f_{\tau }\right)=\exp\left(j\pi f_{\tau }^{2}\;/\;\gamma \right)$ yields the signal after range compression, which is

(3) $$SS\left(f_{\tau },f_{\eta };R_{T}\right)=\exp\left(-j\frac{2\pi f_{\eta }}{v}X_{T}\right)\exp\left[-j\frac{4\pi R_{T}}{c}\sqrt{{\left(f_{c}+f_{\tau }\right)}^{2}-{\left(\frac{cf_{\eta }}{2v}\right)}^{2}}\right].$$

Then, the EOK algorithm is used for RCMC, which can be expressed as

(4) $$f_{r}≜\sqrt{{\left(f_{c}+f_{\tau }\right)}^{2}-{\left(\frac{cf_{\eta }}{2v}\right)}^{2}}-\sqrt{f_{c}^{2}-{\left(\frac{cf_{\eta }}{2v}\right)}^{2}},$$

where $f_{r}$ is the new range frequency. Substituting Equation (4) into Equation (3), we can obtain the signal after RCMC as follows:

(5) $$SS\left(f_{r},f_{\eta };R_{T}\right)=\exp\left(-j\frac{2\pi f_{\eta }}{v}X_{T}\right)\exp\left(-j\frac{4\pi f_{r}}{c}R_{T}\right)\exp\left[-j\frac{4\pi R_{T}}{c}\sqrt{f_{c}^{2}-{\left(\frac{cf_{\eta }}{2v}\right)}^{2}}\right].$$

The first and the second terms in Equation (5) denote the azimuth and range locations of the targets, respectively. In addition, the third term is the azimuth compression term. Multiplying Equation (5) with the azimuth compression function

(6) $$H_{ac}\left(f_{\eta };R_{T}\right)=\exp\left[j\frac{4\pi R_{T}}{c}\sqrt{f_{c}^{2}-{\left(\frac{cf_{\eta }}{2v}\right)}^{2}}\right]$$

and performing 2D inverse Fourier transform, we can obtain the imaging results as

(7) $$I\left(x,r\right)=A\left(x,r\right)\mathrm{sinc}\left[\frac{B_{a}}{v}\left(x-X_{T}\right)\right]\mathrm{sinc}\left[\frac{2B}{c}\left(r-R_{T}\right)\right],$$

where A(x,r) is the amplitude, *x* is the azimuth coordinate, and *r* is the range coordinate. $B_{a}$ and *B* denote the Doppler band and range frequency band, respectively.

For the sake of clarity, $ss(\tau ,\eta ;R_{T})$ is called the raw echo domain signal, $SS(f_{\tau },f_{\eta };R_{T})$ is called the Doppler domain signal, and I(x,r) is called the image domain signal. The imaging procedure transforms the raw echo domain signal into an image domain by RCMC in the Doppler domain. In practice, the imaging procedure is performed in a discrete format.The under-sampling in Doppler domain will lead to the azimuth ambiguity in the image domain. In the following sections, the reason of the azimuth ambiguity is discussed in detail, and a novel method for resolving this problem is proposed.

## 3. The Azimuth Ambiguity

For the sake of clarity, some notations used in the full aperture processing method are listed in Table 1. As the azimuth ambiguity is discussed here, we only consider the signal in azimuth dimension in follows. The azimuth discrete format of full aperture signal in raw echo domain, Doppler domain and image domain can be respectively rewritten as

(8) $$ss_{d,full}\left(\tau ,n;R_{T}\right)=\exp\left\{j\pi \gamma {\left[\tau -\frac{2R(n\Delta \eta -T_{a}\;/\;2;R_{T})}{c}\right]}^{2}\right\}\exp\left[-j\frac{4\pi f_{c}}{c}R\left(n\Delta \eta -T_{a}\;/\;2;R_{T}\right)\right],$$

where Δη=1/PRF, 0≤n≤N−1, and Ta=NΔη.

(9) $$SS_{d,full}\left(f_{r},m;R_{T}\right)=\exp\left[-j\frac{2\pi \left(m\Delta f_{\eta }-PRF\;/\;2\right)}{v}X_{T}\right]\exp\left(-j\frac{4\pi f_{r}}{c}R_{T}\right),$$

where $\Delta f_{\eta }=1\;/\;T_{a}$, 0≤m≤N−1, and $PRF=N\Delta f_{\eta }$.

(10) $$I_{d,full}\left(p,r\right)=A\left(p,r\right)\mathrm{sinc}\left[\frac{B_{a}}{v}\left(p\Delta x-L_{a}\;/\;2-X_{T}\right)\right]\mathrm{sinc}\left[\frac{2B}{c}\left(r-R_{T}\right)\right],$$

where Δx=v/PRF, 0≤p≤N−1, and $L_{a}=N\Delta x$.

**Figure 2:**
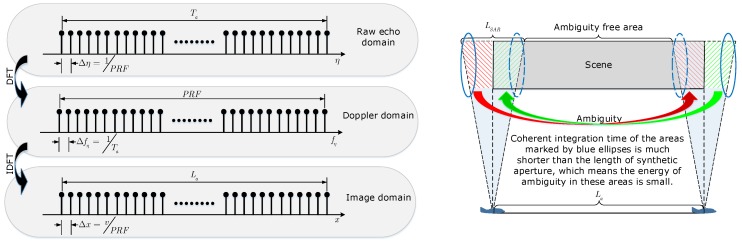
Full aperture processing. (**Left**): relationship between different signal spaces. (**Right**): full aperture observation geometry.

In full aperture processing, *N*-points discrete Fourier transform (DFT) is performed to transform the signal from raw echo domain into Doppler domain, In addition, *N*-points inverse DFT (IDFT) is performed to transform the signal from Doppler domain into the image domain. The relationship between different signal spaces is illustrated as the left graphic in Figure 2. $L_{a}$ is the extension of imaging results in azimuth dimension. By using the property of DFT that the signal length in image domain is determined by the sampling interval in Doppler domain, $L_{a}$ can also be expressed as

(11) $$L_{a}=v\;/\;\Delta f_{\eta }=vT_{a},$$

which means that the maximum azimuth extension of imaging results is equal to the length of full aperture and determined by the $\Delta f_{\eta }$ or $T_{a}$ when performing *N*-points DFT or IDFT. However, from the SAR observation geometry shown as the right graphic in Figure 2, it can be found that the maximum azimuth extension of the scene illuminated by the radar beam is $\left(L_{a}+L_{SAR}\right)$, which is larger than the azimuth extension of imaging results $L_{a}$. Thus, the areas marked by the red and green color in Figure 2 will be folded into image baseband and the azimuth ambiguity occurs. The ambiguity period is equal to $L_{a}$ determined by the $\Delta f_{\eta }$. Correspondingly, the imaging results with ambiguities should be modified as

(12) $$I_{am,full}\left(p,r\right)=\left\{\begin{array}{cc} I_{d,full}\left(p,r\right)+A_{1,full}\left(p,r\right) & 0\leq p\Delta x\leq r\tan\frac{\theta_{b}}{2},\\ I_{d,full}\left(p,r\right) & r\tan\frac{\theta_{b}}{2}<p\Delta x<L_{a}-r\tan\frac{\theta_{b}}{2},\\ I_{d,full}\left(p,r\right)+A_{2,full}\left(p,r\right) & L_{a}-r\tan\frac{\theta_{b}}{2}\leq p\Delta x\leq L_{a}, \end{array}\right.$$

where $\theta_{b}$ is the beam width that is equal to 16${}^{\circ }$ in our LF UWB SAR system, and

(13) $$A_{1,full}\left(p,r\right)=A^{\prime }\left(p+\frac{L_{a}}{\Delta x},r\right)\mathrm{sinc}\left[\frac{B_{a}^{\prime }}{v}\left(p\Delta x+L_{a}\;/\;2-X_{T}\right)\right]\mathrm{sinc}\left[\frac{2B}{c}\left(r-R_{T}\right)\right]$$

denotes the ambiguity area marked by the red color in Figure 2. In addition,

(14) $$A_{2,full}\left(p,r\right)=A^{\prime }\left(p-\frac{L_{a}}{\Delta x},r\right)\mathrm{sinc}\left[\frac{B_{a}^{\prime }}{v}\left(p\Delta x-3L_{a}\;/\;2-X_{T}\right)\right]\mathrm{sinc}\left[\frac{2B}{c}\left(r-R_{T}\right)\right]$$

denotes the ambiguity area marked by the green color in Figure 2. In Equation (12), there is an assumption used that $L_{a}>2r\tan\theta_{b}\;/\;2=L_{SAR}$.

Due to the coherent integration time of the ambiguity areas being shorter than one synthetic aperture time, the amplitude of $A_{1,full}\left(p,r\right)$ and $A_{2,full}\left(p,r\right)$ is small as well as the Doppler bandwidth $B_{a}^{\prime }$. In the full aperture processing, the margins of the scene are discarded after the imaging procedure because of the ambiguities and the worse azimuth resolution, and the ambiguity free area shown in Figure 2 is retained.

**Figure 3:**
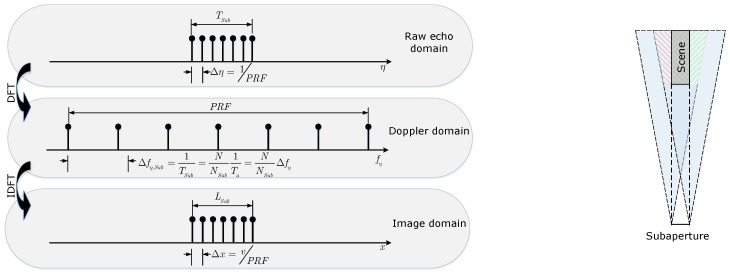
Subaperture processing. (**Left**): relationship between different signal spaces; (**Right**): subaperture observation geometry; in this case, there is no ambiguity free area.

Similarly, the above discussions about ambiguity in full aperture processing is also suitable for the subaperture processing. Notations used in the subaperture processing method are listed in Table 2. Replacing the full aperture notions in Equations (8)–(12) by the subaperture notations, we can obtain the corresponding expressions of the subaperture processing. The relationship between different signal spaces and the observation geometry of subaperture are shown in Figure 3. Compared with the full aperture processing, the sampling interval $\Delta f_{\eta ,Sub}$ is larger than $\Delta f_{\eta }$, which can be expressed as

(15) $$\Delta f_{\eta ,Sub}=\frac{N}{N_{Sub}}\Delta f_{\eta },$$

and $L_{Sub}$ can be expressed as

(16) $$L_{Sub}=v\;/\;\Delta f_{\eta ,Sub}=vT_{Sub}.$$

The larger $\Delta f_{\eta ,Sub}$ is, the smaller $L_{Sub}$ is. The smaller $L_{Sub}$ means the smaller ambiguity free area; even all scenes are contaminated by ambiguities, shown as the right graphic in Figure 3. In real-time SAR imaging processing, the subaperture method is inevitable to fulfill the memory requirement. In theory, every subaperture image is contaminated by ambiguities. Thus, the ambiguities must be suppressed firstly before combining the subaperture images into the full scene image. In the next section, an extended subaperture method is proposed to eliminate the azimuth ambiguities in subaperture images.

## 4. Description of the Extended Subaperture Imaging Method

To eliminate the effect of azimuth ambiguities, an extended subaperture imaging approach based on the extension of the subaperture echo data before RCMC operation is proposed in this paper. Similarly, notations used in the extended subaperture method are listed in Table 3.

**Figure 4:**
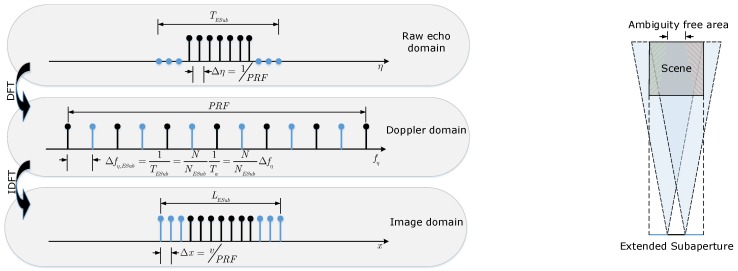
The extended subaperture processing. (**Left**): relationship between different signal spaces; (**Right**): extended subaperture observation geometry.

Compared with the standard subaperture imaging approach, the proposed method first extends the length of subaperture along the azimuth direction by zero-padding on both sides of the subaperture data. After zero-padding in raw echo domain, the sampling interval of the Doppler frequency can be expressed as

(17) $$\Delta f_{\eta ,ESub}=\frac{N}{N_{ESub}}\Delta f_{\eta },$$

which is smaller than $\Delta f_{\eta ,Sub}$ as the $N_{ESub}>N_{Sub}$. Namely, the zero-padding operation in raw echo domain improves the sampling rate in Doppler domain. Then, the azimuth extension of imaging results in the extended subaperture method is

(18) $$L_{ESub}=v\;/\;\Delta f_{\eta ,ESub}=vT_{ESub},$$

which is larger than $L_{Sub}$. The relationship between different signal spaces and the observation geometry of the extended subaperture is shown in Figure 4. Compared with the subaperture processing, the maximum azimuth extension of the scene illuminated by the radar beam is still $\left(L_{sub}+L_{SAR}\right)$, but the azimuth extension of imaging results is enlarged from $L_{sub}$ to $L_{ESub}$, which means a larger ambiguity free area in the extended subaperture method. The ambiguities are mainly located in the extended part and can be cut off after performing the RCMC operation. Finally, the ambiguities are suppressed, and the quality of obtained image is improved.

In an extended subaperture method, the length of ambiguity free area *L* can be expressed as

(19) $$L=3L_{ESub}-2L_{SAR}.$$

In extended subaperture processing, it is preferred that *L* is larger than $L_{Sub}$, so the length of the extended subaperture is constrained by

(20) $$L_{ESub}>\frac{2L_{SAR}}{3}+\frac{L_{Sub}}{3}.$$

Equation (20) also gives the length of zero-padding. As mentioned above, the amplitudes of ambiguities are small due to the short coherent integration time. Thus, the constraint on $L_{ESub}$ can be loosened in practice.

**Figure 5:**
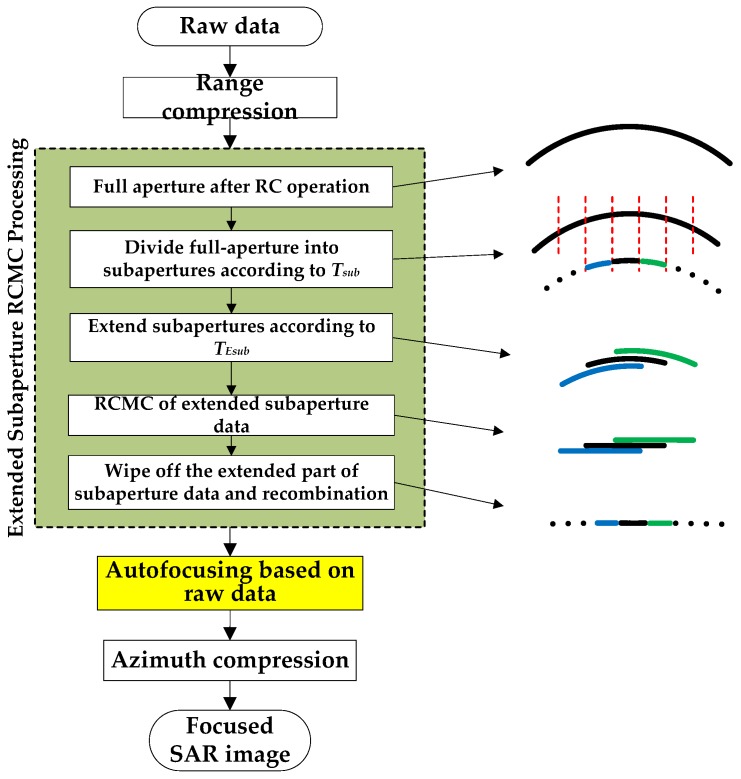
The flow diagram of the extended subaperture imaging processing for UWB SAR.

Figure 5 shows the flow diagram of proposed extended subaperture imaging processing for LF UWB SAR. Compared with Figure 1, there are two additional steps, i.e., “Extended the subaperture data” and “Wipe off the extended subaperture data”, which are performed before and after the step of “RCMC processing of the extended subaperture data”, respectively. In practical applications, there are two ways to extend the subaperture length in the azimuth direction before RCMC operation. One way is to perform zero-padding on both sides of the subaperture data. Another way is the overlapping subaperture method. In fact, these two methods are identical in essence, and people can choose the suitable method for real application according to their specific requirement, and the extra length of each extended subaperture is $T_{ESub}-T_{Sub}$.

## 5. Experiment Results

### 5.1. Simulated Experiment

To evaluate the azimuth ambiguity suppression performance of the proposed extended subaperture method, a simulated experiment was first carried out. The simulation parameters are listed in Table 4. The EOK algorithm [4] was used in both the subaperture and the extended subaperture methods, and the focused quality of the target located at the center range was analyzed in detail.

**Figure 6:**
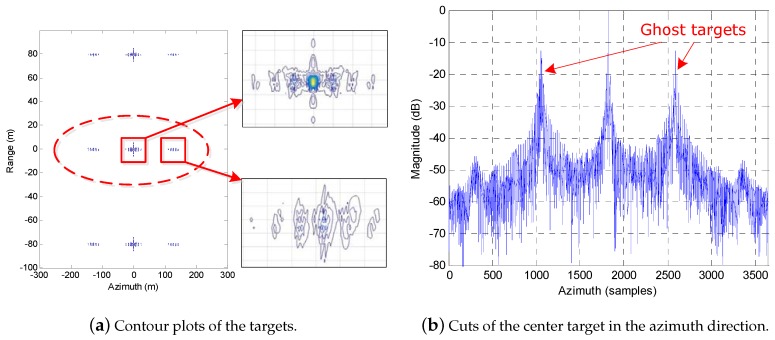
The results obtained by the subaperture imaging method.

In the simulated experiment, there are three targets in the scene located along the range direction with the same azimuth positions. In the imaging processing, the full-aperture echo is divided into nine nonoverlapping subapertures echo data. Figure 6a shows the imaging results processed by the traditional subaperture imaging method, and we can find that two ghost targets present on both sides of each true target, which are induced by the azimuth ambiguities in the traditional subaperture method. Figure 6b shows the azimuth profile of the center target, in which the maximum magnitude of the two ghost targets is about −12 dB. Furthermore, the azimuth distance between the ghost target and the true target is equal to the length of subaperture, which is consistent with the theory analysis presented in Section 3.

**Figure 7:**
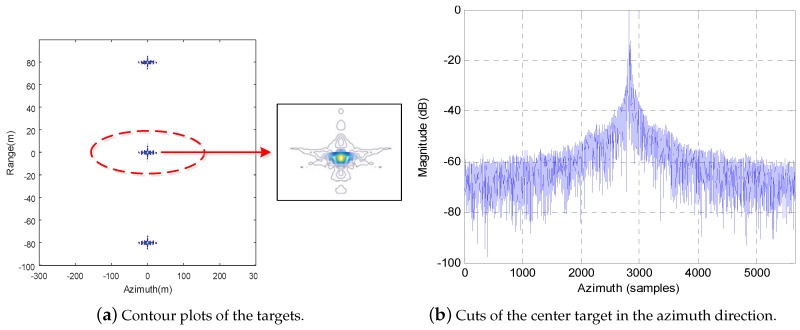
The results obtained by the proposed extended subaperture imaging method.

Comparing with the results shown in Figure 6 and Figure 7 gives the results obtained by the proposed extended subaperture imaging method, in which the full-aperture echo is also firstly divided into nine nonoverlapping subapertures data after RC operation, and then each piece of subaperture data is extended double the amount on both sides along the azimuth direction before RCMC operation. Observing the imaging results shown in Figure 7, it is easy to find that the azimuth ambiguity problem is effectively resolved, and the ghost targets are completely removed from the imaging results. Correspondingly, the focused quality is greatly improved.

Furthermore, Figure 6 and Figure 7 also show the enlarged contour plots of the real target and the ghost target obtained by the different imaging methods. We can find that, compared with the subaperture method, our extended subaperture method not only eliminates the ghost targets, but also improves the focused quality of the real target.

### 5.2. Raw Data Experiment

**Figure 8:**
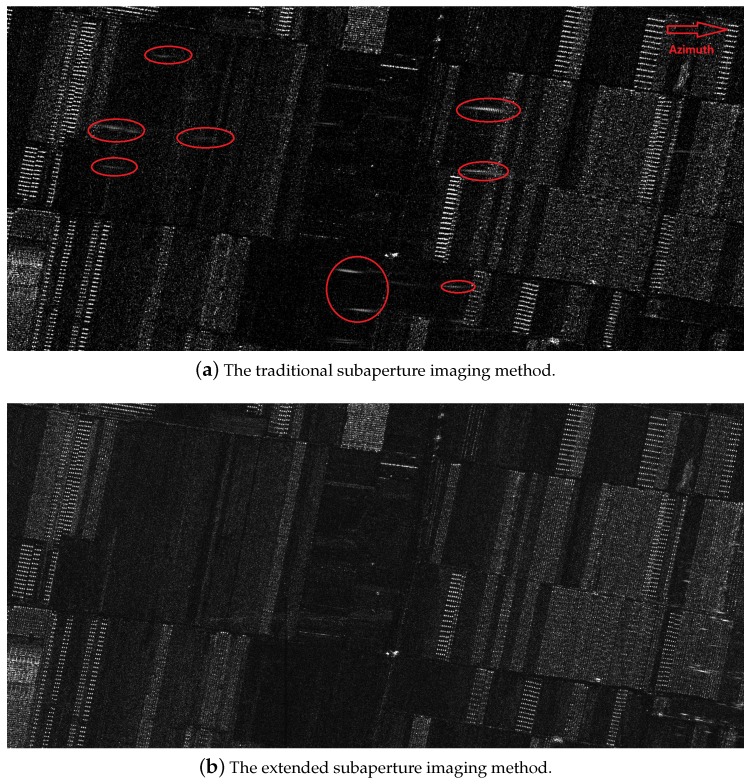
The imaging results of real LF UWB SAR data by using the different methods.

To further evaluate the performance of the proposed extended subaperture imaging method, an experiment with raw data was also carried out. The raw data was acquired by an airborne LF UWB SAR system operated on P band, the signal bandwidth is 200 MHz, the center slant range is 10 km, and the spatial resolution is about 1 m. Due to the large antenna beam width in azimuth direction (as large as 16${}^{\circ }$), the azimuth ambiguity problem is severe. The traditional subaperture imaging method and the extended subaperture imaging method were adopted for the raw data processing, respectively. Figure 8 shows the imaging results obtained by the different subaperture imaging methods. In Figure 8a, due to azimuth ambiguities induced by the traditional subaperture imaging, there are many ghost targets (such as the targets circled by the yellow dashed ellipse) in the obtained image, which degrade the focused quality, and seriously affect the following SAR image processing, such as target detection, image interpretation, and so on. Comparatively, Figure 8b shows the image obtained by the proposed extended subaperture imaging method. We can find that all the ghost targets generated by the azimuth ambiguities disappeared, and the image with better quality is obtained. The imaging results based on the raw data prove the correctness and effectiveness of our proposed method.

## 6. Conclusions

This paper first introduces the subaperture imaging method that always adopted in LF UWB SAR raw data processing, and the reason for azimuth ambiguities in the traditional subaperture imaging method was deeply analyzed in theory. To resolve this problem, a novel method based on extending the subaperture echo data along the azimuth direction before the RCMC is proposed, which can significantly suppress the azimuth ambiguities, remove the ghost targets, and finally obtain the well-focused image. The proposed method has simple implementation and excellent performance on resolving azimuth ambiguities problem, which is validated by the simulated and real LF UWB SAR data. It should be noted that, in the real-time processing, the data volume and computational load will be increased due to the extension of subaperture data, which contradicts the degree of suppressing the azimuth ambiguities. Therefore, people should select the proper extended length of subaperture in practice according to their specific requirement.

## Figures and Tables

**Table 1:** Notations used in the full aperture processing.

Notation	Description
Δη	the sampling interval of the slow time η
$\Delta f_{\eta }$	the sampling interval of the Doppler frequency $f_{\eta }$
Δx	the sampling interval of the azimuth location in image domain
$T_{a}$	the length of the full aperture time, i.e., $-T_{a}\;/\;2\leq \eta \leq T_{a}\;/\;2$
PRF	the pulse repetition frequency, i.e., $-PRF\;/\;2\leq f_{\eta }\leq PRF\;/\;2$
$L_{a}$	the azimuth length of imaging results, i.e., $-L_{a}\;/\;2\leq x\leq L_{a}\;/\;2$
$L_{SAR}$	the length of one synthetic aperture
$B_{a}$	the Doppler band
*N*	the number of sampling points in full aperture

**Table 2:** Notations used in the subaperture processing.

Notation	Description
$\Delta f_{\eta ,Sub}$	the sampling interval of the Doppler frequency $f_{\eta }$
$T_{Sub}$	the length of the subaperture time, i.e., $-T_{Sub}\;/\;2\leq \eta \leq T_{Sub}\;/\;2$
$L_{Sub}$	the azimuth length of imaging results, i.e., $-L_{Sub}\;/\;2\leq x\leq L_{Sub}\;/\;2$
$B_{a,Sub}$	the Doppler band of subaperture
$N_{sub}$	the number of sampling points in subaperture

**Table 3:** Notations used in the extended subaperture processing.

Notation	Description
$\Delta f_{\eta ,ESub}$	the sampling interval of the Doppler frequency $f_{\eta }$
$T_{ESub}$	the length of the extended subaperture time, i.e., $-T_{Sub}\;/\;2\leq \eta \leq T_{Sub}\;/\;2$
$L_{ESub}$	the azimuth length of imaging results, i.e., $-L_{Sub}\;/\;2\leq x\leq L_{Sub}\;/\;2$
$B_{a,ESub}$	the Doppler band of the extended subaperture
$N_{Esub}$	the number of sampling points after zero-padding

**Table 4:** Simulation parameters.

Parameters	Values
Operated frequency	P Band
Bandwidth	200 MHz
Sampling frequency	250 MHz
Pulse Repetition Frequency (PRF)	500 Hz
The reference range	5 Km
Azimuth resolution	1 m
